# Comparative Genomic and Secretomic Analysis Provide Insights Into Unique Agar Degradation Function of Marine Bacterium *Vibrio fluvialis* A8 Through Horizontal Gene Transfer

**DOI:** 10.3389/fmicb.2020.01934

**Published:** 2020-08-11

**Authors:** Chunsheng Li, Chi Li, Laihao Li, Xianqing Yang, Shengjun Chen, Bo Qi, Yongqiang Zhao

**Affiliations:** ^1^Key Laboratory of Aquatic Product Processing, Ministry of Agriculture and Rural Affairs, National R&D Center for Aquatic Product Processing, South China Sea Fisheries Research Institute, Chinese Academy of Fishery Sciences, Guangzhou, China; ^2^Co-Innovation Center of Jiangsu Marine Bio-industry Technology, Jiangsu Ocean University, Lianyungang, China

**Keywords:** complete genome, secretome, agarase, horizontal gene transfer, *Vibrio*

## Abstract

Agarose-oligosaccharide production from agar degradation by agarase exhibits lots of advantages and good application prospects. In this study, a novel agar-degrading bacterium *Vibrio* sp. A8 was isolated from a red algae in the South China Sea. The whole genome sequencing with comparative genomic and secretomic analysis were used to better understand its genetic components about agar degradation. This strain exhibited good agarase production in artificial seawater after culture optimization. The complete genome (4.88 Mb) of this strain comprised two circular chromosomes (3.19 and 1.69 Mb) containing 4,572 protein-coding genes, 108 tRNA genes and 31 rRNA genes. This strain was identified as *Vibrio fluvialis* A8 by comparative genomic analysis based on genome phylogenetic tree and average nucleotide identity (ANI) similarity. Different from other 20 similar strains including three strains of the same species, *V. fluvialis* A8 possessed unique agar degradation ability with four β-agarases (GH50) and one α-1,3-L-NA2 hydrolase (GH117) due to the horizontal gene transfer. Secretomic analysis showed that only β-agarase (gene 3152) was abundantly expressed in the secretome of *V. fluvialis* A8. This agarase had a good substrate specificity and wide work conditions in complex environments, suggesting its potential application for agarose-oligosaccharide production.

## Introduction

Agar oligosaccharides (AOS) produced by agarase has a good application prospect. Agar, a marine polysaccharide producing from red algae, is widely used in food industry, pharmaceutical industry and biological engineering (Won-Jae et al., 2013). As the main component of agar, agarose is composed of 3,6-anhydro-L-galactose (L-AHG) and D-galactose (D-Gal) bonded alternately by α-1,3 and β-1,4 linkages (Araki, 1956). Agarose-oligosaccharides, including AOS and neoagaroligosacchrides (NAOS), are agarose degradation products, which exhibit various physiological functions, such as antioxidant (Kang et al., 2014), anti-inflammatory activity (Enoki et al., 2010), whitening effect (Kim et al., 2017), immune modulation (Mussatto and Mancilha, 2007), and antitumor activity (Bhattarai and Kashyap, 2016). There are two methods to produce agarose-oligosaccharides, including acid hydrolysis and agarase biodegradation. Compared with acid hydrolysis, agarase biodegradation can be easy to control agarose-oligosaccharide types, and produce the products with fixed polymerization degrees in an environmentally friendly way without damage of structure (Xu et al., 2018). Therefore, it is very important to develop agarases in the industrial production of agarose-oligosaccharides.

Most agarases are produced by marine gram-negative bacteria including *Agarivorans* (Zhang et al., 2019), *Pseudoalteromonas* (Wang et al., 2018; Li et al., 2019), *Microbulbifer* (Ma et al., 2019; Zhu et al., 2019), *Alteromonas* (Seo et al., 2014), etc., *Vibrio* is also an important genus of marine gram-negative bacteria to produce agarases, and various strains have been reported about their agarase production characteristics, such as *Vibrio* sp. JT0107 (Jam et al., 2005), *Vibrio* sp. CN41 (Liao et al., 2011), *Vibrio* sp. ZC-1 (Su et al., 2019), *Vibrio* sp. EJY3 (Yu et al., 2020), *Vibrio* sp. PO-303 (Dong et al., 2007), and *Vibrio* sp. V134 (Zhang and Sun, 2007). The agarases produced by these strains have been well characterized and classified. However, the genomic information and specific taxonomic status of these strains in *Vibrio* remain poorly understood. Here, we isolated and characterized a marine bacterium *Vibrio* sp. A8 with high agar degradation ability from a red algae in the South China Sea. This strain was identified as *Vibrio fluvialis* and showed 99.38% similarity with *V. fluvialis* NBRC103150 by 16S rRNA gene sequencing. To date, only three genome sequencing projects of *V. fluvialis* have been completed according to the NCBI Genome database. However, interestingly, no agarase genes have been found in these genomes based on the gene sequences and annotation data. Therefore, it is significantly useful and interesting to study whether *Vibrio* sp. A8 belongs to *V. fluvialis*, whether this strain can produce agarase, and why this strain possesses unique agar degradation ability.

In this study, the complete genome of novel agar-degrading bacterium *Vibrio* sp. A8 was sequenced using a combination of next-generation sequencing and third-generation sequencing technology. The microbial functions were annotated and analyzed against the gene databases to investigate the genetic elements related to agar degradation. Comparative genomic analysis between *Vibrio* sp. A8 and 20 similar strains in *Vibrio* was performed to ascertain the taxonomy position of this strain and to explain whether these bacteria had a common ability of agar degradation. Secretomic analysis using nanoLC-MS/MS was performed to study the molecular properties of agarase in the secretome of this strain, followed by the analysis of the biochemical properties of purified agarase. This study provides information about novel agarase in *Vibrio* and fills in the gap on the genomic information and taxonomy position of the agar-degrading strain in this genus, which is valuable for better understanding the agar degradation pathway in the biofilm of red algae surface, and providing insights into biotechnological applications of agarase for production of agarose-oligosaccharides.

## Materials and Methods

### Isolation and Biochemical Identification of Strain A8

Marine bacterium strain *Vibrio* sp. A8 with efficient agar degradation ability was isolated from the rotten red algae (*Gracilaria lemaneiformis*) in the South China Sea. Firstly, 5.0 g rotten algae was added into 50 mL sterilized saline water. After sufficient vortex oscillation, the solution was spread on LB ager plates (1% peptone, 0.5% yeast extract powder, 1% NaCl and 1.8% agar; pH 7.0) using gradient dilution. After cultivation at 37°C, the strains with concave around their colonies were selected and inoculated on fresh LB ager plates for 48 h at 37°C. The plates were then stained with Lugol’s iodine solution (5% I_2_ and 10% KI). Colonies with a clear zone formed by agar degradation were determined as agar-degrading strains. The strain with the highest agar degradation ability was maintained on LB agar slant (1% peptone, 0.5% yeast extract powder, 1% NaCl, and 1.8% agar; pH 7.0) and was used for further study.

Biochemical identification of *Vibrio* sp. A8 was carried out using the VITEK 2 GN test kit (BioMérieux) according to the manufacturer’s instructions.

### Agarase-Producing Condition Optimization

*Vibrio* sp. A8 was first pre-cultured by transferring a loopful of slant culture to liquid LB medium (1% peptone, 0.5% yeast extract powder, and 1% NaCl; pH 7.0) and incubated at 37°C and 180 r/min for 24 h. Thereafter, the strain was transferred to 50 mL liquid agarase-producing medium (0.2% yeast extract powder and 0.2% agar in artificial seawater; pH 7.0) in 250 mL Erlenmeyer flask.

Unless otherwise stated in the agarase-producing experiments, the inoculation size of the strain after pre-culture was 2%, the pH value of the medium was 7.0, the culture temperature was 37°C, and the culture time was 24 h. Different carbon sources (0.2% galactose, 0.2% glucose, 0.2% sucrose, 0.2% agar, 0.2% agar + 0.2% galactose, 0.2% agar + 0.2% glucose, or 0.2% sucrose + 0.2% agar) were first added in the agarase-producing medium, and then the effects of different concentrations of the optimum carbon source on the agarase activity in the medium was studied. The optimum nitrogen source for the agarase production was selected by changing the yeast extract powder in the medium into other nitrogen sources (0.2% peptone, 0.2% NH_4_NO_3_, 0.2% (NH_4_)_2_SO_4_, 0.2% NH_4_Cl, or 0.2% NH_4_Cl + 0.2% yeast extract powder). The effects of NaCl concentrations (0–2.5%), pH value (4–9), temperature (15–40°C), and culture time (0–48 h) on the agarase activity in the medium were studied to determine the optimum culture conditions of *Vibrio* sp. A8.

The agarase activity was determined by measuring the release of the reducing sugars using the 3,5-dinitrosalicylic acid (DNS) method (Miller, 1959). Briefly, the medium (100 μL) after cultivation was incubated with 0.2% melted agar (2 mL) at 40°C for 30 min. The solution was then placed in boiling water for 5 min to stop the enzymatic reaction. Subsequently, the DNS reagent (2 mL) was added to the reaction solution which was heated in a boiling water bath for 5 min. When dropped to room temperature, the mixture was diluted with distilled water to 25 mL, and the released reducing sugars were quantified spectrophotometrically at 520 nm. One unit of agarase activity was defined as the amount of enzymes that released 1 μg of reducing sugars per minute under these experimental conditions.

### Whole Genome Sequencing, Assembly and Annotation

The genomic DNA from *Vibrio* sp. A8 was extracted and purified using a Wizard Genomic DNA Purification Kit (Promega). The whole genome was sequenced using a combination of PacBio RS II SMRT and Illumina sequencing platforms. For Illumina sequencing, the genomic DNA sample (over 1 μg) was sheared into 400–500 bp fragments using a Covaris M220 Focused Acoustic Shearer (Covaris). Illumina sequencing libraries were got from these fragments using the NEXTflex^TM^ Rapid DNA-Seq Kit (Bioo Scientific), and were then used for paired-end Illumina sequencing (2 × 150 bp) on an Illumina HiSeq × Ten machine (Illumina). For Pacific Biosciences sequencing, an aliquot of 15 μg DNA was spun in a Covaris g-TUBE (Covaris) at 6,000 r/min for 60 s. DNA fragments were then purified, end-repaired and ligated with SMRTbell sequencing adapters (Pacific Biosciences). The resulting sequencing library was purified three times using Agencourt AMPure XP beads (Beckman Coulter Genomics) and sequenced on one SMRT cell (Pacific Biosciences) using standard methods.

The complete genome was assembled using both the PacBio and Illumina reads (Feng et al., 2019). Briefly, the PacBio reads were assembled into a contig using HGAP and Canu. The circular step was checked and finished manually, generating a complete genome with seamless chromosomes and plasmids. The Illumina reads was used for the error correction of the PacBio assembly results using Pilon. The CDS, tRNA, and rRNA was, respectively predicated by Glimmer version 3.02, tRNA-scan-SE version 2.0 and Barrnap version 0.8. The predicted CDSs were annotated from NR, Swiss-Prot, Pfam, GO, COG, KEGG, and CAZyme database using sequence alignment tools such as BLAST, Blast2go, eggNOG, Diamond, and HMMER. Each set of query proteins were aligned with the databases, and annotations of the best-matched subject (*E*-value < 10^–5^) were used for gene annotation. The gene islands were predicted using the Island Viewer (Bertelli et al., 2017). The circular genomic plot of PMTB2.1 was plotted by Circos (Song H. et al., 2016).

The agarase and NAOS hydrolase gene sequences were compared with the gene sequences available from the NCBI database. Phylogenetic tree of these gene sequences were performed using MEGA based on the maximum-likelihood method after multiple alignments of the sequences by Clustal X (Larkin et al., 2007). Numbers represented the frequencies with which the tree topology was replicated after 1000 bootstrap iterations.

### Comparative Genomic Analysis

Genomic data of the similar Vibrio species analyzed in this study were obtained from the NCBI. The homologous genes were analyzed using OrthoMCL (Li et al., 2003) with the following parameters: E-Value, 1e^–5^; Percent Identity Cutoff, 0; Markov Inflation Index, 1.5. Phylogenetic analysis was performed by RA × ML (Stamatakis, 2006) after multiple alignments of single copy of homologous genes using Clustal X (Larkin et al., 2007). To compare the genetic relationship among Vibrio species, the average nucleotide identity (ANI) value was calculated using Jspecies (Richter and Rossello-Mora, 2009).

### Secretomic Analysis

Label-free quantitative LC-MS/MS was performed to study the soluble extracellular proteins of Vibrio sp. A8. The extracellular proteins were isolated by ammonium sulfate fractionation (30∼60% ammonium sulfate saturation), redissolved in phosphate buffer solution (0.05 mol/L, pH 7.0), and then desalted by dialysis bag. The Bradford method (Bradford, 1976) was used to determine the protein concentration of sample. The protein sample (120 μg) was digested using trypsin (Promega) at 37°C overnight. Equal volume of 1% formic acid was mixed with digested sample and centrifuged at 12000 × g for 5 min at room temperature. The supernatant was slowly loaded to the C18 desalting column, washed by 1 mL of washing solution (0.1% formic acid and 4% acetonitrile) three times, and then eluted twice using 0.4 mL of elution buffer (0.1% formic acid and 75% acetonitrile). The eluents were lyophilized and reconstituted in 10 μL of 0.1% formic acid. Peptide samples were injected and analyzed using a Q Exactive HF-X mass spectrometer (Thermo) coupled with nanoEASY-nLC 1200 UHPLC (Thermo). Peptides were first trapped onto a home-made C18 Nano-Trap column (2 cm × 75 μm, 3 μm) and then eluted onto a home-made analytical column (15 cm × 150 μm, 1.9 μm) via a 60 min gradient of 2–80% acetonitrile with 0.1% formic acid at a flow rate of 600 nL/min (0–2 min: 4.8%; 2–49 min: 4.8–8%; 49–52 min: 4.8–24%; 52–54 min: 24–28%; 54–55 min: 28–40%; 55–60 min: 40–80%). MS full scan ranged from m/z 350 to 1500 with a target value of 3,000,000 at a resolution of 60,000 (at m/z 200). The top 40 precursors of the highest abundant in the full scan were fragmented using higher energy collisional dissociation, and analyzed in MS/MS with an automatic gain control target value of 50,000 and normalized collision energy of 27%. Raw MS/MS data were searched against the genome of Vibrio sp. A8 obtained in this study using Proteome Discoverer 2.2 (Thermo). For abundance calculation, mass spectrometric signal intensities of peptide precursor ions belonging to each protein were divided by the total abundance of all detected proteins. The mass spectrometry proteomics data have been deposited to the ProteomeXchange Consortium via the PRIDE (Perez-Riverol et al., 2019) partner repository with the dataset identifier PXD019302.

Phylogenetic tree of agarases based on their amino acid sequences was performed using MEGA based on the neighbor-joining method after multiple alignments of the sequences by Clustal X (Larkin et al., 2007). The sequences of agarases that were similar to that in Vibrio sp. A8 were obtained from UniProt database. Numbers represented the frequencies with which the tree topology was replicated after 1000 bootstrap iterations.

### Agarase Purification and Properties

The extracellular proteins were isolated using ammonium sulfate fractionation (30∼60% ammonium sulfate saturation), redissolved in phosphate buffer solution (0.05 mol/L, pH 7.0), and then desalted by dialysis bag. The agarase purification was performed by DEAE Sephadex anion-exchange chromatography. The proteins were eluted by NaCl solution at 0.3 mol/L. The eluent collected from the protein peak was tested for the agarase activity. The eluent with the agarase activity was used for the study of agarase properties.

Unless otherwise stated in the study of agarase properties, the diluted enzyme solution (100 μL) was incubated with 0.2% melted agar (2 mL) at 40°C and pH 7.0 for 30 min, in which condition the agarase activity was set as 100%. The agarase solution was incubated with 2 mL of 2 g/L carrageenan, algin, chitosan, cellulose, or amylum to study the substrate specificity of agarase. One unit of enzyme activity was defined as the amount of enzymes that released 1 μg of reducing sugars per minute under these experimental conditions using DNS method as mentioned previously. To study the optimal reaction temperature, the agarase activity was measured at different temperatures (25, 30, 35, 40, 45, 50 and 55°C). The optimal pH of the agarase was assayed in a pH range of 4.0–9.0 in 20 mM Na_2_HPO_4_-citrate buffer (pH 4.0–6.0), 20 mM phosphate buffer (pH 7.0–8.0), and 20 mM glycine–NaOH buffer (pH 9.0). The effects of different metal ions (NaCl, FeCl_2_, FeCl_3_, CaCl_2_, CuSO_4_, KCl or MgCl_2_) at 1 mmol/L on the agarase activity were also studied. The same amount of agarase solution was placed in the water bath at 35, 40, 45, and 50°C, respectively for 0–60 min to study the agarase thermostability. The data were fitted to first order plots, and plots of ln(RA) (residual activity) versus time were constructed to calculate k_d_. The half-life of the enzyme (t_1/2_) was calculated using the following equation:

$${\text{T}}_{1/2}=\frac{\ln2}{k_{\text{d}}}$$

where *t*_1/2_ is the time required for the residual activity reducing to half, and *k*_d_ is the deactivation rate constant.

## Results and Discussion

### Agarase-Producing Condition Optimization of Strain A8

**FIGURE 1:**
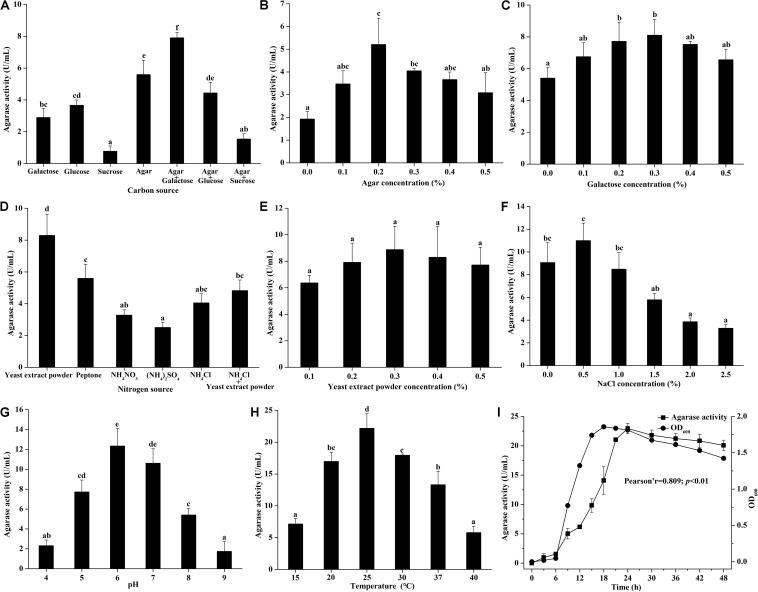
Effect of **(A)** carbon source (with 0.2% yeast extract powder), **(B)** agar concentration (with 0.2% yeast extract powder), **(C)** galactose concentration (with 0.2% yeast extract powder and 0.2% agar), **(D)** nitrogen source (with 0.2% agar and 0.3% galactose), **(E)** yeast extract powder concentration (with 0.2% agar and 0.3% galactose), **(F)** NaCl concentration (with 0.2% agar, 0.3% galactose, and 0.3% yeast extract powder), **(G)** pH (with 0.2% agar, 0.3% galactose, 0.3% yeast extract powder, and 0.5% NaCl), **(H)** temperature (with 0.2% agar, 0.3% galactose, 0.3% yeast extract powder, and 0.5% NaCl at pH 6.0), and **(I)** culture time (with 0.2% agar, 0.3% galactose, 0.3% yeast extract powder, and 0.5% NaCl at 25°C and pH 6.0) on the agarase activity in artificial seawater cultivation with strain A8. Bars labeled with the same letter are not statistically different (*p* < 0.05) tested by one-way ANOVA and multiple comparison Tukey test. Pearson correlation analysis was tested between agarase activity and OD_600_ at different culture time.

The effects of medium components and culture conditions on the agarase production of *Vibrio* sp. A8 are shown in Figure 1. Different carbon sources showed obviously different effects on the agarase activity in the medium (Figure 1A). When glucose, galactose, sucrose or agar was added to the medium alone, the agarase activity was the highest in the presence of agar. However, the agarase activity decreased after glucose and sucrose were added to the medium, possibly because the co-existence of glucose or sucrose reduced the utilization of agar by this strain, thus inhibiting the secretion of agarase. The addition of galactose enhanced the agarase activity with the agar as the only carbon source, probably resulting from the improved strain growth by galactose. Therefore, the agar and galactose were chosen as the carbon sources of the agar-producing medium. The effects of agar and galactose concentrations on the agarase activity in the medium were, respectively studied. As shown in Figures 1B,C, the agarase activity in the medium first increased and then decreased with the increasing agar or galactose concentrations. The optimum agar and galactose concentration was respectively 0.2 and 0.3%.

Different agarase activities was found in the medium with the addition of different nitrogen source (Figure 1D). Organic nitrogen source was more suitable for the agarase production of *Vibrio* sp. A8 than inorganic nitrogen source. The agarase activity was highest when yeast extract powder was the only nitrogen source in the medium. The agarase activity was not significantly different at various yeast extract powder concentrations (0.1–0.5%) (Figure 1E). The highest agarase activity was observed at 0.3% yeast extract powder.

Based on artificial seawater, the different concentrations of NaCl were added to determine the optimum osmotic pressure for the agarase production of *Vibrio* sp. A8 (Figure 1F). The agarase activity was improved by 0.5% NaCl but was significantly inhibited by 2.0–2.5% NaCl. This might be because the excessive osmotic pressure inhibited the strain growth, leading to the decrease of its agarase production. Different pH values had great influence on the agarase activity in the medium (Figure 1G). The optimum pH was 6.0, at which the agarase activity was 12.35 U/mL. Similar to pH, the culture temperature showed significant influence on the agarase production of strain A8 (Figure 1H). The optimum culture temperature was 25°C, at which the agarase activity was as high as 22.19 U/mL.

The effect of culture time on the agarase production of *Vibrio* sp. A8 is shown in Figure 1I. The agarase activity increased slightly at 0–6 h, markedly rised at 6–24 h, and then reached equilibrium after 24 h. The growth curve was in accordance with the agarase-producing curve. Pearson correlation analysis showed that the growth of strain A8 was significantly correlated with agarase activity (*p* < 0.01), indicating that the positive influence of strain growth on the agarase production.

The optimum medium for agarase production of *Vibrio* sp. A8 was determined as 0.2% agar, 0.3% galactose, 0.3% yeast extract powder, and 0.5% NaCl in artificial seawater, and the optimum culture conditions were determined as temperature at 25°C, pH at 6.0 and incubation time for 24 h. Under these conditions, the agarase activity of the fermentation broth was 22.28 U/mL, which was 314% higher than that before optimization.

### Genome Features of Strain A8

**FIGURE 2:**
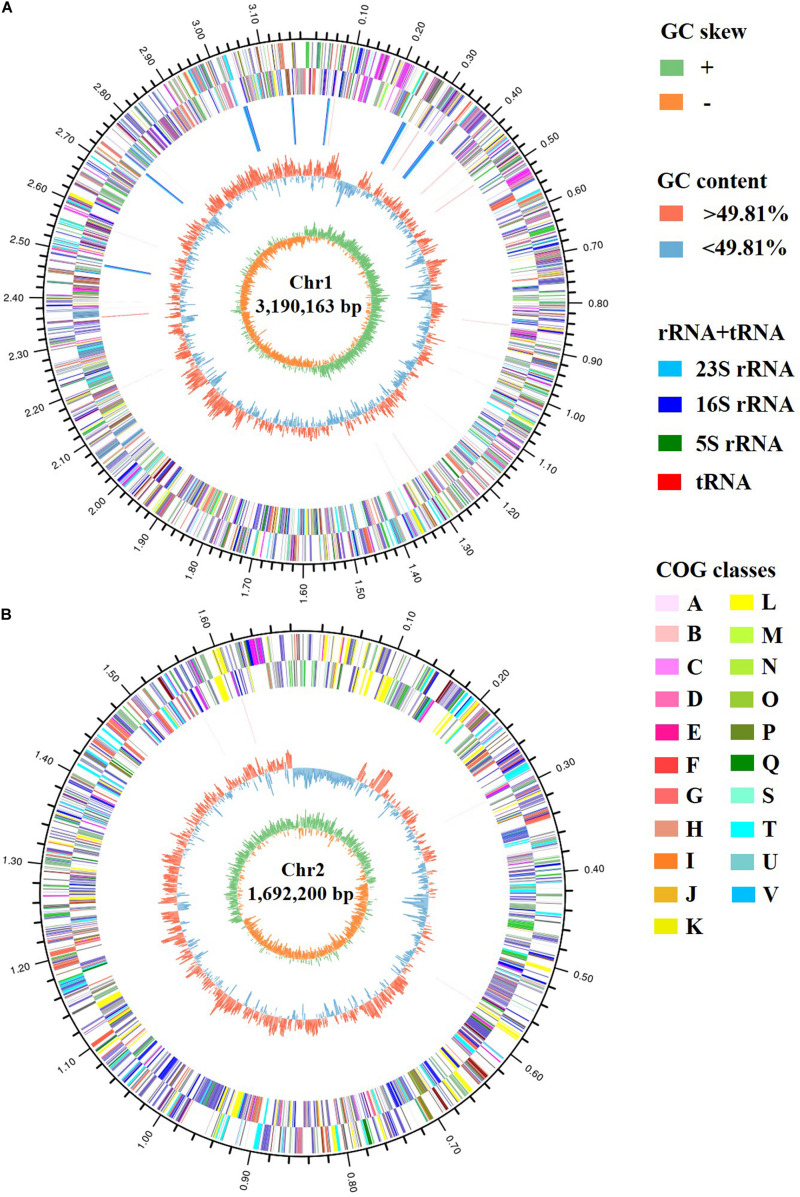
Circular maps of **(A)** Chr1 and **(B)** Chr2 of strain A8. The circles represent (from inner to outer): (1) GC skew; (2) GC content; (3) rRNA and tRNA; (4) COG assignments for CDSs on the reverse strand; (5) COG assignments for CDSs on the forward strand; (6) scale in Mb.

The genomic features of *Vibrio* sp. A8 are presented in Table 1. The complete genome of *Vibrio* sp. A8 was 4,882,363 bp (49.81% G+C) in length, composed of two circular chromosomes, Chr1 (3,190,163 bp, 49.89% G+C) (Figure 2A) and Chr2 (1,692,200 bp, 49.67% G+C) (Figure 2B), and no plasmid was detected. The genome contained 4,572 protein-coding genes, 108 tRNA genes and 31 rRNA genes, and all rRNA genes were located in Chr1. There were 3,401 genes (74.39%) assigned to the GO terms, including 2,633 genes in biological process, 2,737 genes in molecular function, and 1,823 genes in cellular component. The most abundant GO annotation included the metabolic process (1,929 genes), membrane (1,159 genes), and catalytic activity (1,845 genes) in respective category (Supplementary Figure S1). There were 3,975 genes (86.94%) assigned to the COG terms, and high percentage of genes was classified into the amino acid transport and metabolism (318 genes), transcription (305 genes), inorganic ion transport and metabolism (240 genes), energy production and conversion (238 genes), and carbohydrate transport and metabolism (230 genes), as well as the function unknown (1,112 genes) (Supplementary Figure S1). In addition, 2,645 genes (57.85%) were allocated to the KEGG pathways, in which the carbohydrate metabolism (242 genes), global and overview maps (237 genes), amino acid metabolism (224 genes) and membrane transport (222 genes) were the predominant pathways (Supplementary Figure S1).

**TABLE 1:** Genomic features of strain A8.

Feature	Value
Genome size (bp)	4,882,363
DNA G+C	49.81%
Chromosome	2
Plasmid	0
Protein-coding genes	4,572
tRNAs	108
rRNAs	31
Tandem repeats	34
Genes assigned to GO	3,401
Genes assigned to COG	3,975
Genes assigned to KEGG	2,645
Genes with Pfam domains	3,874
Genes coding for CAZymes	112
Genes related to secondary metabolism	113
Genes related to virulent factor	536
Genes related to antibiotic resistance	288
Genes coding for secretory proteins	314
Genes coding for transport proteins	944
Genes coding for transmembrane proteins	1,028
Gene islands	11

### Identification of Strain A8

**FIGURE 3:**
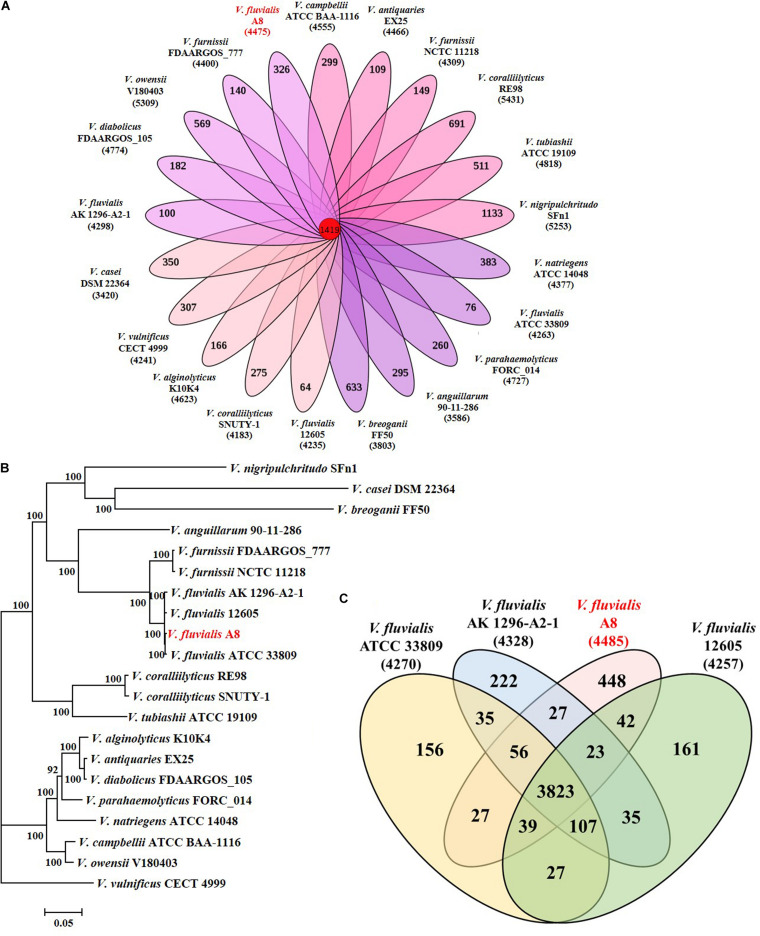
Phylogeny of *Vibrio* species based on single-copy core orthologs. **(A)** Core homologous genome (1419 genes) was computed by the genome of strain A8 along with 20 complete *Vibrio* genomes obtained from public database. **(B)** Phylogenetic analysis was performed by RA × ML after multiple alignments of single copy of homologous genes using Clustal X. **(C)** Venn diagram showing homologous and unique genes among *V. fluvialis* strains.

Strain A8 was initially identified as *Vibrio* sp. A8 based on 16S rRNA gene sequence. To determine the exact species of this strain, the comparative genomic analysis between A8 and 20 similar strains in *Vibrio* was studied. Among the 21 *Vibrio* strains, 1,419 homologous genes were found and constructed the core genome (Figure 3A). The genome phylogenetic tree was then constructed based on the core homologous genome (Figure 3B). The phylogenetic tree was capable of grouping various strains of a single species into a single cluster. Strain A8 clustered with *V. fluvialis* ATCC 33809, *V. fluvialis* 12605, and *V. fluvialis* AK 1296-A2-1, suggesting that these four strains might harbor similar biological properties. The biochemical identification and characterization observations for strain A8 were made on VITEK 2 GN system (Supplementary Table S1). Strain A8 was identified as *V. fluvialis* with 90% of probability. On the basis of above results, the strain A8 could be determined as *V. fluvialis* and was renamed as *V. fluvialis* A8. The ANI similarity analysis also proved that strain A8 belonged to *V. fluvialis*. It was reported that the strains could be considered as the same prokaryotic species when the ANI value was over 96% (Richter and Rossello-Mora, 2009). As shown in Table 2, intraspecies similarity of *V. fluvialis* was higher than 97%. The highest similarity with *V. fluvialis* A8 was *V. fluvialis* ATCC 33809, reaching 98.5%, which was consistent with the result of phylogenetic tree. Although 3,823 homologous genes were found among these *V. fluvialis* strains, there were still 448 unique genes in *V. fluvialis* A8 (Figure 3C).

**TABLE 2:** Heatmap of the ANI value between the conserved regions in the genomes of strains in *Vibrio*.

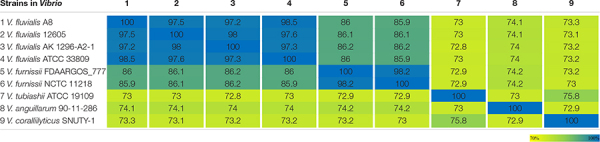

*Species on the first column and first row of the table are represented in the same numeric order. The numbers show the percentage similarity between the conserved regions of the genomes, where the colors vary from yellow (low similarity) to blue (high similarity).*

### Genes Related to Agar Degradation in *Vibrio fluvialis* A8

**FIGURE 4:**
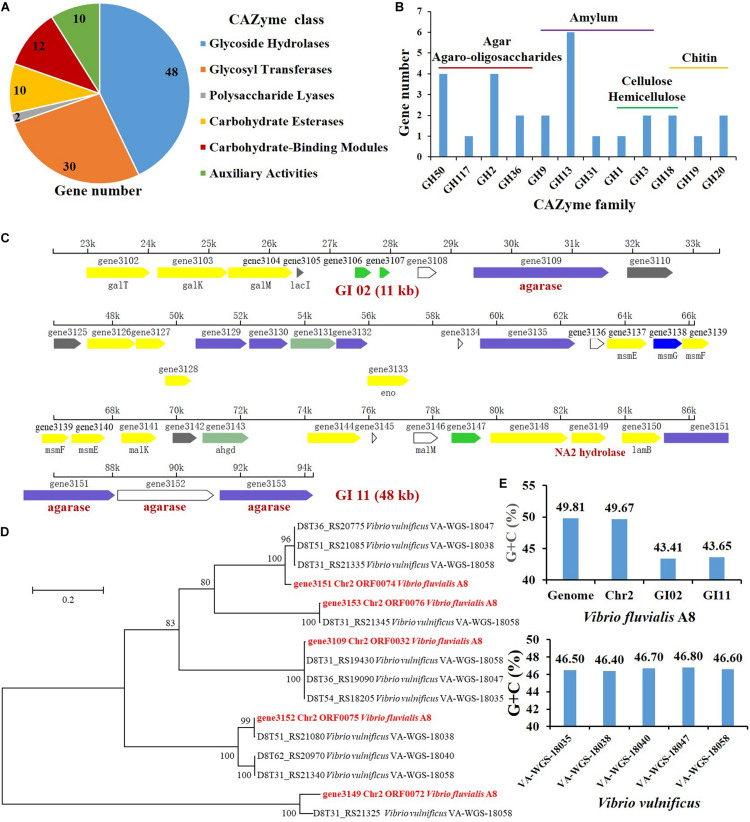
Genes related to agar degradation in the genome of V. fluvialis A8. **(A)** Number of genes in each CAZyme class. **(B)** Number of genes related to polysaccharide degradation in GHs. Genomic islands (GI) contained genes related to agar degradation: **(C)** gene3149 coding for α-1,3-L-NA2 hydrolase, and gene3151, gene3152, and gene3153 coding for β-agarase were in GI 11; **(D)** gene3109 coding for β-agarase was in GI 02. **(E)** The G+C content in the GI 02, GI 11, Chr2, and genome of V. fluvialis A8 as well as in the genomes of V. vulnificus strains.

Carbohydrate-active enzymes (CAZymes) that are responsible for the complex carbohydrate metabolism, mainly include the recognition (carbohydrate-binding module, CBMs), synthesis (glycosyltransferases, GTs), and degradation (glycoside hydrolases, GHs; polysaccharide lyases, PLs; carbohydrate esterases, CEs; and enzymes of auxiliary activities, AAs) of the carbohydrates (Huang et al., 2018). There were 112 genes coding for CAZymes in the genome of *V. fluvialis* A8 (Table 1). This strain had a high fraction of carbohydrate-degrading enzymes, especially GHs (48 genes) which could hydrolyze various polysaccharides, including agar, agaro-oligosaccharides, amylum, cellulose, hemicellulose, and chitin (Figure 4).

Agarases are classified into α-agarase (E.C. 3.2.1.158) and β-agarase (E.C. 3.2.1.81) according to their cleavage position on agarose: α-agarase catalyzes the α-1,3 linkage to produce AOS, while β-Agarase produces NAOS by split of β-1,4 linkage (Fu and Kim, 2010). Agarases are grouped into GHs based on the sequence similarity, e.g., β-Agarase grouping into the GH16, GH50, GH86, and GH118 families and α-agarase only grouping into the GH96 family (Su et al., 2019). In this study, a total of four genes coding for β-agarase which all belonged to GH50 family, were found in the genome of *V. fluvialis* A8. Among the β-agarase genes, the gene3151, gene3152, and gene3153 arranged in order from 85221 to 94263 on the forward strand of Chr2, while the gene3109 was located on the reverse strand of Chr2 from 31608 to 29374 (Table 3). NAOS can be further catalyzed by α-NAOS hydrolase (EC 3.2.1.159) which has been classified into GH 117 family and can recognize neoagarobiose (NA2), neoagarotetraose (NA4), and neoagarohexaose (NA6) as substrate (Ariga et al., 2014; Watanabe et al., 2016). In the genome of *V. fluvialis* A8, the gene3149 coding for α-1,3-L-NA2 hydrolase was classified into GH 117 family. This enzyme can hydrolyze NA2 and produce L-AHG and D-Gal (Watanabe et al., 2016). It was reported that β-galactosidase in *Vibrio* sp. EJY3 could act on AOS to release D-Gal (Lee et al., 2014). There were four genes of β-galactosidase and two genes of α-galactosidase in the genome of *V. fluvialis* A8, which might contribute to its ability of agar degradation. The abundant genes of agarase, NAOS hydrolase and galactosidase in *V. fluvialis* A8 offer an enormous advantage in the saccharification of agar in red algae for its use as a biomass feedstock for the production of fermentable sugar.

**TABLE 3:** Genes related to degradation of agar and relative compounds in the genome of *V. fluvialis* A8.

Gene ID	Location	Strand	Start	End	Gene length	Protein length	Annotation	GH family
gene3109	Chr2	−	31608	29374	2235	744	β-agarase	GH50
gene3151	Chr2	+	85221	88079	2859	952	β-agarase	GH50
gene3152	Chr2	+	88164	91154	2991	996	β-agarase	GH50
gene3153	Chr2	+	91348	94263	2916	971	β-agarase	GH50
gene3149	Chr2	−	83414	82332	1083	360	α-1,3-L-NA2 hydrolase	GH117
gene0378	Chr1	+	392498	393304	807	268	β-galactosidase	GH2
gene1513	Chr1	−	1594442	1591350	3093	1030	β-D-galactosidase	GH2
gene3148	Chr2	−	82225	79805	2421	806	β-galactosidase	GH2
gene4670	Chr2	+	1647838	1649307	1470	489	β-galactosidase	GH2
gene1669	Chr1	−	1769658	1768117	1542	513	α-galactosidase	GH36
gene3155	Chr2	−	98435	96333	2103	700	α-galactosidase	GH36

To study whether the strains closely related to *V. fluvialis* A8 shared the common ability of agar degradation, the agar-degrading genes of these strains were analyzed by comparative genomic analysis (Table 4). Interestingly, although the *Vibrio* strains exhibited the genes coding for galactosidase, the homologs for agarase and NA2 hydrolase were not observed in all other tested strains, even in the same species *V. fluvialis* 12605, *V. fluvialis* AK 1296-A2-1, and *V. fluvialis* ATCC 33809. This suggested that *V. fluvialis* A8 was probably different from other similar strains and possessed the unique agar degradation ability. Although the agar-degrading genes were not found in the tested genomes, another *Vibrio* sp. strain EJY3 exhibited similar agarolytic system with *V. fluvialis* A8 and also could produce four β-agarases (GH50) and one α-NAOS hydrolase (GH117) (Yu et al., 2020). Horizontal gene transfer, also known as lateral gene transfer, refers to nonsexual transmission of genetic material between unrelated genomes (Oliveira et al., 2017). As the most important form of horizontal gene transfer, genomic island (GI) contains numerous genes associated with a variety of biological functions (Godde et al., 2018). It has been reported that horizontal gene transfer in the bacteria affected the phenotypes such as thermotolerance and multidrug resistance of closely related strains (Hegstad et al., 2020; Matsutani et al., 2020). Horizontal gene transfer also gives bacteria unique polysaccharide degradation ability. *Alteromonas macleodii* 83-1 harbored an alginolytic system within a 24 kb GI, which comprised five putative alginate lyases and other CAZymes (Neumann et al., 2015). The marine bacterium *Pseudoalteromonas haloplanktis* ANT/505 acquired a GI with a functional pathway for pectin catabolism, including two multi-modular pectate lyases, PelA and PelB (Hehemann et al., 2017). A novel agarase gene *aga1* in an inland soil agar-degrading bacterium *Paenibacillus* sp. SSG-1 might be horizontally transferred from marine bacteria via human microbiota (Song T. et al., 2016). In the genus *Vibrio*, horizontal gene transfer mainly contributes to the virulence of strains (Le Roux and Blokesch, 2018; Deng et al., 2019). However, few studies of horizontal gene transfer related to agar degradation were reported in the genus *Vibrio*. In the genome of *V. fluvialis* A8, there were 11 GIs, in which 474 genes located, accounting for over 10% of total protein-coding genes. Interestingly, all the GIs were located in the Chr2 of this strain. The genes coding for β-agarase and α-1,3-L-NA2 hydrolase were all contained in the GIs: the gene3151, gene3152, gene3153, and gene3149 were in the GI 11 (Figure 4C), while the gene3109 were in the GI 02 (Figure 4D). These results proved that these genes related to agar degradation were obtained from other species through horizontal gene transfer. The gene sequence similarity was further studied to determine the probable source using the phylogenetic tree analysis (Figure 4D). The gene3109, gene3151, gene3152, gene3153, and gene3149 were found most similar to that in *Vibrio vulnificus* VA-WGS-18058 (percent identity with 99.78%), *V. vulnificus* VA-WGS-18047 (percent identity with 96.08%), *V. vulnificus* VA-WGS-18038 (percent identity with 99.77%), *V. vulnificus* VA-WGS-18058 (percent identity with 99.90%), and *V. vulnificus* VA-WGS-18058 (percent identity with 91.20%), respectively. Furthermore, the G+C content in the GI 02 and GI 11 was 43.41 and 43.65%, respectively, which was obviously lower than that in the genome (49.81% G+C) and Chr2 (49.67% G+C) of *V. fluvialis* A8, but much closer to that in the genome of *V. vulnificus* strains (46.40–46.80% G+C) (Figure 4E). These results indicated that these genes might come from these *V. vulnificus* strains through horizontal gene transfer.

**TABLE 4:** Genes involved in degradation of agar and relative compounds in the genomes of strains in *Vibrio* based on homologous gene analysis.

	Strains in *Vibrio*	Agar degradation (gene numbers)		
		
		Agarase	Neoagarobiose hydrolase	Galacto sidase
1	*V. fluvialis* A8	+ (4)	+ (1)	+ (6)
2	*V. fluvialis* 12605	−	−	+ (4)
3	*V. fluvialis* AK 1296-A2-1	−	−	+ (4)
4	*V. fluvialis* ATCC 33809	−	−	+ (4)
5	*V. furnissii* FDAARGOS_777	−	−	+ (4)
6	*V. furnissii* NCTC 11218	−	−	+ (5)
7	*V. tubiashii* ATCC 19109	−	−	+ (4)
8	*V. anguillarum* 90-11-286	−	−	+ (3)
9	*V. coralliilyticus* SNUTY-1	−	−	+ (1)
10	*V. coralliilyticus* RE98	−	−	+ (5)
11	*V. antiquaries* EX25	−	−	+ (3)
12	*V. parahaemolyticus* FORC_014	−	−	+ (3)
13	*V. alginolyticus* K10K4	−	−	+ (2)
14	*V. natriegens* ATCC 14048	−	−	+ (4)
15	*V. diabolicus* FDAARGOS_105	−	−	+ (3)
16	*V. campbellii* ATCC BAA-1116	−	−	+ (2)
17	*V. owensii* V180403	−	−	+ (6)
18	*V. breoganii* FF50	−	−	+ (4)
19	*V. vulnificus* CECT 4999	−	−	+ (4)
20	*V. casei* DSM 22364	−	−	+ (4)
21	*V. nigripulchritudo* SFn1	−	−	+ (2)

*+, present; −, absent.*

### Agarase Determination in *Vibrio fluvialis* A8 Based on Secretomic Analysis

Secretomic analysis using nanoLC-MS/MS was performed to study the agarase produced from *V. fluvialis* A8. A total of 1,078 peptides were identified and belonged to 207 proteins. The molecular weight of these proteins ranged from 6.6 to 212.2 kDa, and most of these proteins focused between 30 and 40 kDa (Supplementary Figure S2). The function of proteins with high abundance in the secretome of *V. fluvialis* A8 mainly included three major categories: macromolecule degradation (zinc protease, agarase, metalloprotease, and aminopeptidase), cell components (flagellin protein FlaD, FlaA, and FlaC, and membrane protein), and virulent factor (hemolysin HlyA, thermolabile hemolysin precursor) (Table 5). These proteins provided a great advantage for utilization the nutrients from red algae that helped *V. fluvialis* A8 grow. Among these extracellular proteins, zinc protease coded by gene3819 showed the highest relative abundance reaching 69.49%. A total of eight proteins were found in GHs, in which β-agarase coded by gene3152 had the highest relative abundance reaching 3.04% (Table 5). The 27 peptides of this agarase tested by Label-free quantitative LC-MS/MS were shown in Supplementary Table S2. Interestingly, there were four genes in the genome of *V. fluvialis* A8, among which the gene3152 and gene3153 coding for β-agarases were predicted as secretory proteins with signal peptide (Sec/SPI) using SignalP version 5.0, but only gene3152 was expressed extracellularly. It might be because there was little expression of the gene3153. In addition, the β-agarase encoded by gene3151 had a lipoprotein signal peptide (Sec/SPII), which was also called lipobox. Lipobox played an important role in anchoring the agarases onto the cell surface by acylation (Ekborg et al., 2006; Hutcheson et al., 2011). In this study, different from β-agarase (gene3152) that was secreted into the medium, β-agarase (gene3151) was predicted to be bound to the cell surface, which also contributed to the agar degradation and utilization of *V. fluvialis* A8. The β-agarase (gene3152) in the secretome was about 108.7 kDa with the pI 4.60 (Table 5).

**TABLE 5:** Proteins with the highest abundance and enzymes belonging to the GHs in the secretome of *V. fluvialis* A8.

	Protein ID	Gene ID	Annotation	MW [kDa]	Calculated pI	Relative abundance (%)
Proteins with the	3819	gene3819	Zinc protease	65.3	5.74	69.49
highest abundance	2266	gene2266	Flagellin protein FlaD	40.0	5.01	8.79
	2186	gene2186	Flagellin protein FlaA	36.6	4.96	5.20
	3152	gene3152	β-agarase	108.7	4.60	3.04
	2187	gene2187	Flagellin protein FlaD	40.1	5.14	2.97
	4060	gene4060	Hemolysin HlyA	80.2	5.27	1.69
	4061	gene4061	Thermolabile hemolysin precursor	46.6	5.07	1.03
	2267	gene2267	Flagellin protein FlaC	40.4	5.01	1.02
	3797	gene3797	Metalloprotease	100.7	4.78	0.83
	2482	gene2482	Membrane protein	37.8	4.75	0.83
	4295	gene4295	Aminopeptidase	67.6	4.65	0.81
Enzymes belonging	3152	gene3152	β-agarase	108.7	4.60	3.04
to the GHs	4670	gene4670	β-galactosidase	55.1	6.05	0.03
	4149	gene4149	Chitodextrinase	89.7	4.49	0.13
	3263	gene3263	Chitodextrinase	109.1	4.68	0.10
	0718	gene0718	Chitodextrinase	61.4	4.70	0.21
	1292	gene1292	Pullulanase	212.2	4.61	0.00
	0815	gene0815	Glycosidase	64.8	5.49	0.01
	3730	gene3730	Glycosyl hydrolase	87.1	4.78	0.00

**FIGURE 5:**
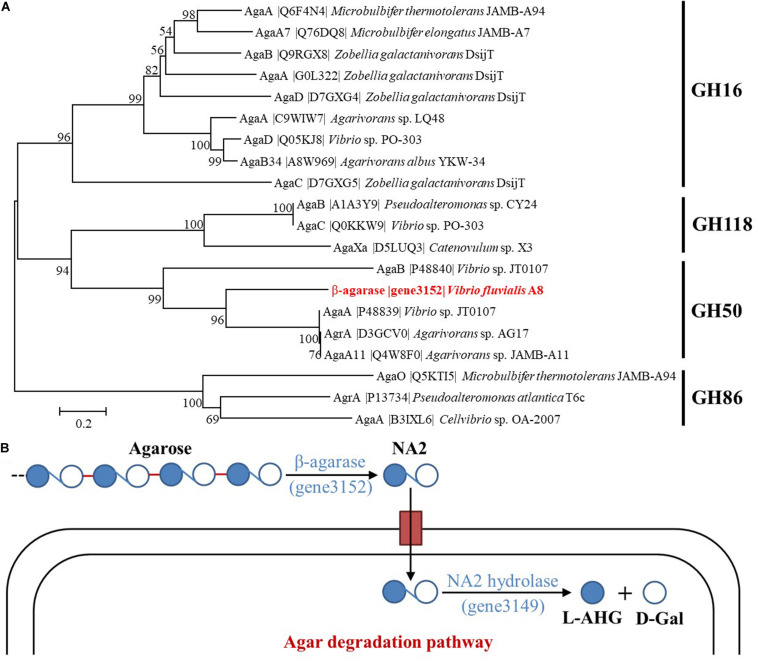
Classification of β-agarase and possible agar metabolic pathways in *V. fluvialis* A8. **(A)** Phylogenetic tree between β-agarase (gene3152) and the characterized agarases obtained from UniProt database. Phylogenetic tree of agarases based on their amino acid sequences was performed by MEGA based on the neighbor joining method after multiple alignments of the sequences by Clustal X. Bootstrap values (expressed as percentages) are given at the branching points. The bar corresponds to a genetic distance of 0.1 substitution per position (10% amino acid sequence difference). **(B)** Illustration for the possible agar metabolic pathways in *V. fluvialis* A8.

Two kinds of β-agarase have been determined in *Vibrio* sp. JT0107, β-agarase A (AgaA) which hydrolyzes agarose and also NA4 to yield NA2, and β-agarase B (AgaB) which hydrolyzes agarose to yield predominantly NA4 and NA6 (Jam et al., 2005). The phylogenetic tree consisting of β-agarase (gene3152) and known characterized agarases proved that the β-agarase secreted from *V. fluvialis* A8 was a member of GH50 (Figure 5A). The β-agarase (gene3152) showed most similar to AgaA (percent identity with 48.41%) from *Vibrio* sp. JT0107, suggesting that it might be classified as AgaA. Therefore, the major agar metabolic pathway in *V. fluvialis* A8 could be speculated: Agar or agarose was first hydrolyzed extracellularly by β-agarase (gene3152) to yield NA2, and after transport into the cells, NA2 was further catalyzed by α-1,3-L-NA2 hydrolase (gene3149) to produce L-AHG and D-GAL for use as a carbon source (Figure 5B).

### Biochemical Properties of Agarase in *Vibrio fluvialis* A8

**FIGURE 6:**
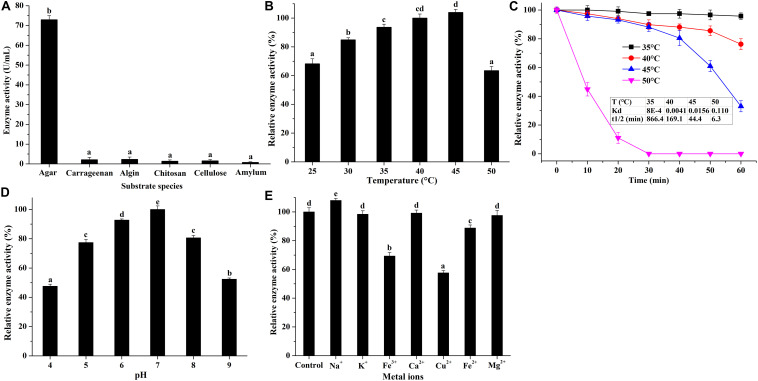
Biochemical properties of purified agarase in *V. fluvialis* A8. **(A)** Substrate specificity of agarase was studied using various substrates, including carrageenan, algin, chitosan, cellulose, and amylum. **(B)** Effects of temperature, **(C)** pH, and **(D)** metal ions on the agarase activity. Bars labeled with the same letter are not statistically different (*p* < 0.05) tested by one-way ANOVA and multiple comparison Tukey test. **(E)** Thermostability of agarase was studied at different temperatures.

After preliminary purification by DEAE Sephadex anion-exchange chromatography (Supplementary Figure S3), the biochemical properties of agarase in the secretome of *V. fluvialis* A8 was studied (Figure 6). The agarase could hardly degrade other polysaccharides such as carrageenan, algin, chitosan, cellulose, and amylum into small-molecule reducing sugars, indicating its good substrate specificity (Figure 6A). The agarase could work over a wide temperature range that its relative activity at 25–50°C maintained over 60% (Figure 6B). The optimum temperature of the agarase was 45°C. This result is consistent with the optimum reaction temperature of agarase from *Cellulophaga omnivescoria* W5C (Ramos et al., 2018) and *Pseudoalteromonas hodoensis* H7 (Chi et al., 2014). The optimum pH of the agarase was 7.0 (Figure 6C). The agarase activity was relatively stable to pH, and the activity could remain over 47% when the pH value was in the range of 4.0–9.0. Such performance could hardly reached by agarase from other bacteria that the activity was significantly inhibited at low pH (Tawara et al., 2015; Chen et al., 2019; Su et al., 2019). The agarase possessed stable activity at high concentration of various metal ions (Figure 6D). Na^+^ significantly enhanced the agarase activity by 8.0%. Mg^2+^, Ca^2+^, and K^+^ had little effect on the agarase activity, while Cu^2+^, Fe^2+^, and Fe^3+^ manifested a strong inhibition of agarase activity. Similar results were found that the activity of agarase was significantly suppressed by Cu^2+^ and Fe^2+^ (Chen et al., 2019; Li et al., 2019). The agarase in *V. fluvialis* A8 showed good thermostability at 35–45°C (Figure 6E). The t_1/2_ of agarase at 35, 40, and 45°C reached 866.4, 169.1, and 44.4 min, respectively. The agarase in *V. fluvialis* A8 was easily inactivated over 50°C and its activity completely lost after 30 min at this temperature. The agarase in *V. fluvialis* A8 possessed more thermostability than agarase rAgaZC-1 and D622G in *Vibrio* sp. ZC-1 whose t_1/2_ was only 3.1 and 6.3 min at 45°C, respectively (Su et al., 2019). In this study, the agarase in *V. fluvialis* A8 had a good substrate specificity, possessed stable activity in the conditions of temperature, pH and various metal ions, and exhibited good thermostability, which suggests that it could be developed as a potential agarase preperation for agarose-oligosaccharide production in future.

## Conclusion

Complete genome of novel agar-degrading bacterium *Vibrio* sp. A8 was sequenced to investigate the genetic elements related to agar degradation. The complete genome was 4.88 Mb with two circular chromosomes (3.19 and 1.69 Mb) and no plasmid. This strain was identified as *V. fluvialis* A8 by genome phylogenetic tree and ANI similarity with biochemical identification. Different from other similar strains including the same species, *V. fluvialis* A8 possessed unique agar degradation ability with 4 β-agarases (GH50) and 1 α-1,3-L-NA2 hydrolase (GH117) due to horizontal gene transfer. Only β-agarase coding by gene3152 was expressed extracellularly based on secretomic analysis. This agarase had a good substrate specificity and stability in complex environments, suggesting its potential application for agarose-oligosaccharide production.

## Data Availability Statement

The datasets presented in this study can be found in online repositories. The names of the repository/repositories and accession number(s) can be found in the article/Supplementary Material.

## Author Contributions

CSL and CL conceived, designed, and performed the experiments. CSL analyzed the data, prepared all tables and figures, and wrote the manuscript. SC and BQ provided assistance with analysis tools. LL, SC, BQ, and YZ critically reviewed and curated the manuscript. CSL and XY were responsible for the project. All authors contributed to the article and approved the submitted version.

## Conflict of Interest

The authors declare that the research was conducted in the absence of any commercial or financial relationships that could be construed as a potential conflict of interest.
