# Supplementary material for: Comparative Genomic and Secretomic Analysis Provide Insights Into Unique Agar Degradation Function of Marine Bacterium Vibrio fluvialis A8 Through Horizontal Gene Transfer
Source: Front Microbiol. 2020 Aug 11;11:1934. doi: 10.3389/fmicb.2020.01934 (PMC7432431; doi:10.3389/fmicb.2020.01934)
Supplement: Supplementary file 1 [file Image_1.PDF]

# *Supplementary Material*

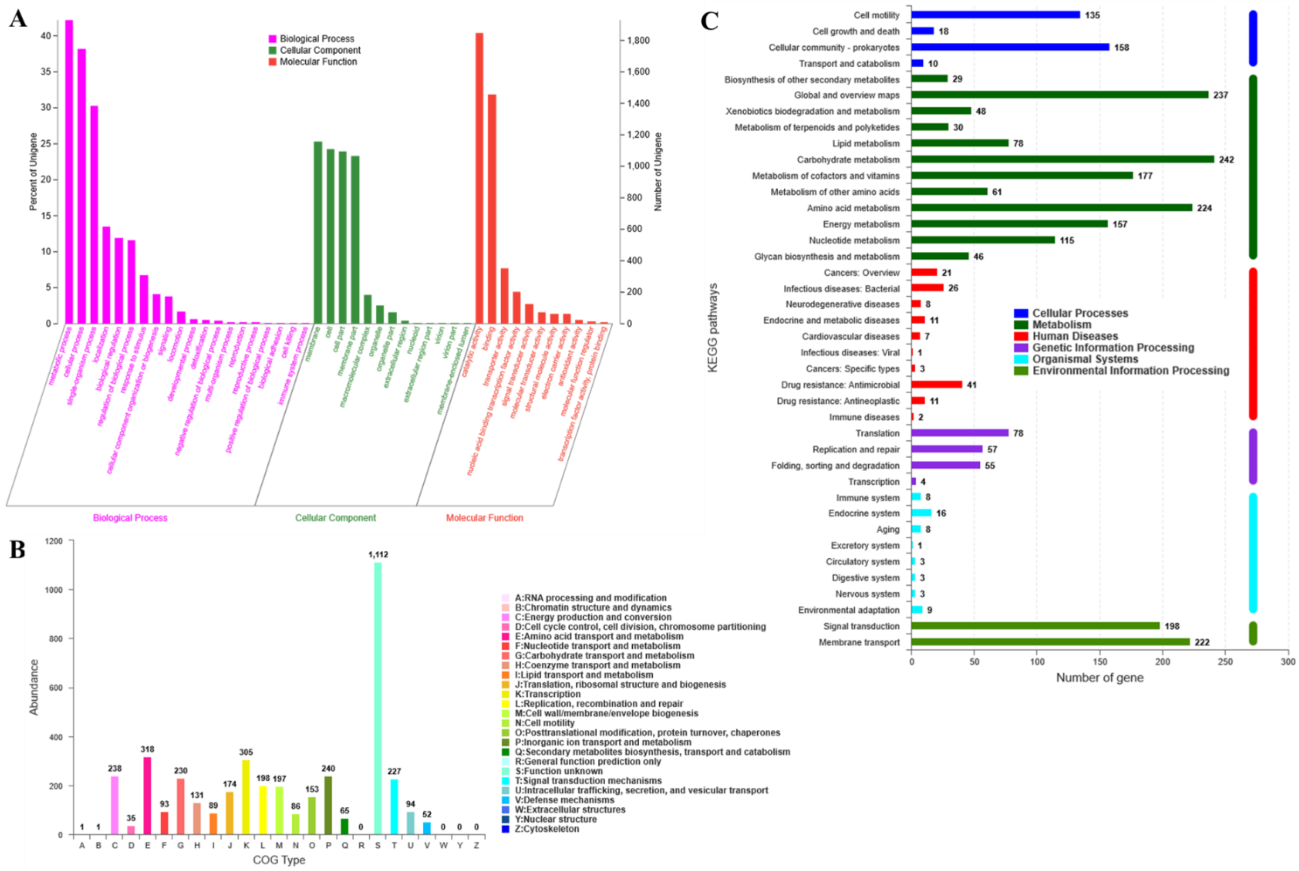


**Supplementary Figure S1** Genes in the genome of *V. fluvialis* A8 assigned to **(A)** GO terms, **(B)** COG terms and **(C)** KEGG pathways.
