# Supplementary material for: Comparative Genomic and Secretomic Analysis Provide Insights Into Unique Agar Degradation Function of Marine Bacterium Vibrio fluvialis A8 Through Horizontal Gene Transfer
Source: Front Microbiol. 2020 Aug 11;11:1934. doi: 10.3389/fmicb.2020.01934 (PMC7432431; doi:10.3389/fmicb.2020.01934)
Supplement: Supplementary file 2 [file Image_2.PDF]

Supplementary Material

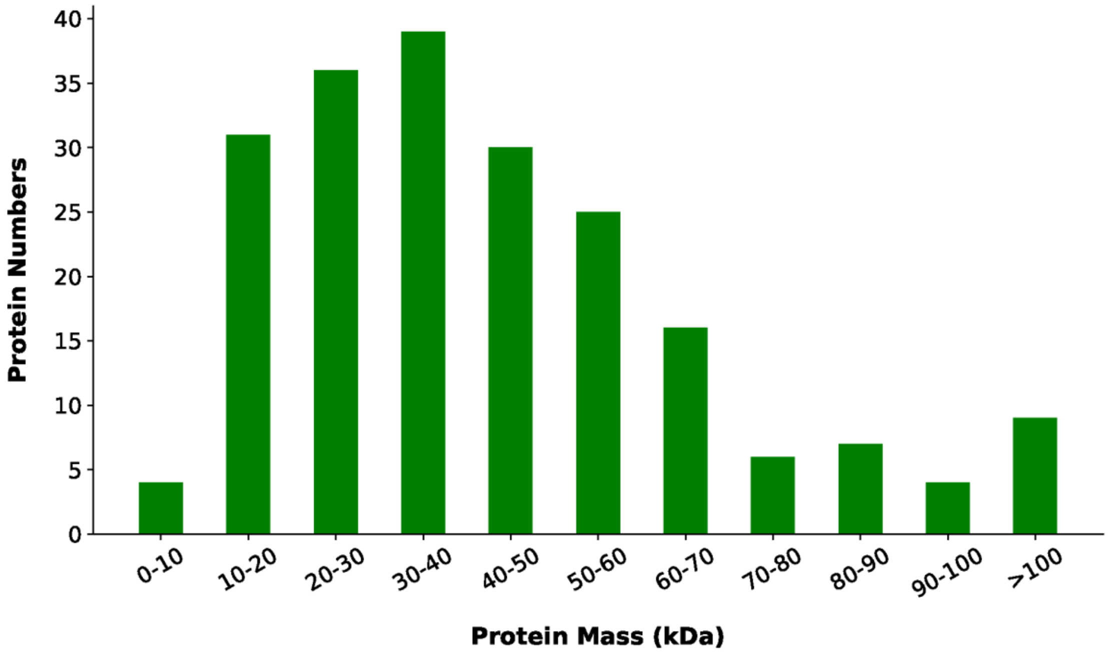

Supplementary Figure S2 Molecular weight (kDa) distribution of proteins identified in the secretome of *V. fluvialis* A8.
