# Supplementary material for: Comparative Genomic and Secretomic Analysis Provide Insights Into Unique Agar Degradation Function of Marine Bacterium Vibrio fluvialis A8 Through Horizontal Gene Transfer
Source: Front Microbiol. 2020 Aug 11;11:1934. doi: 10.3389/fmicb.2020.01934 (PMC7432431; doi:10.3389/fmicb.2020.01934)
Supplement: Supplementary file 3 [file Image_3.PDF]

Supplementary Material

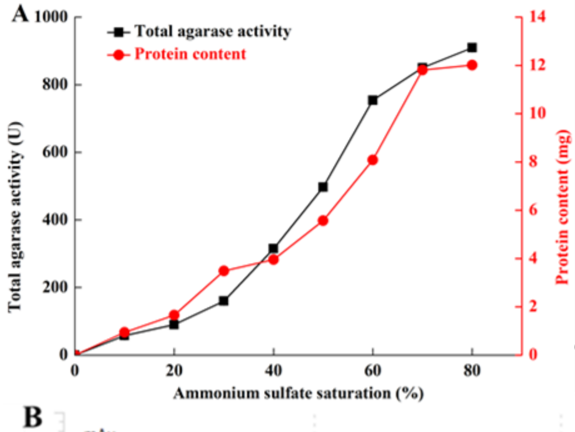

C

	Total protein (mg)	Total agarase activity (U)	Specific activity (U/mg)	Recovery (%)	Purification fold
Fermented medium	92.4	4014	43.41	100	1
Ammonium sulfate precipitation	23.57	1843.5	78.21	45.93	1.81
DEAE anion exchange chromatography	4.31	609.95	141.52	15.21	3.26

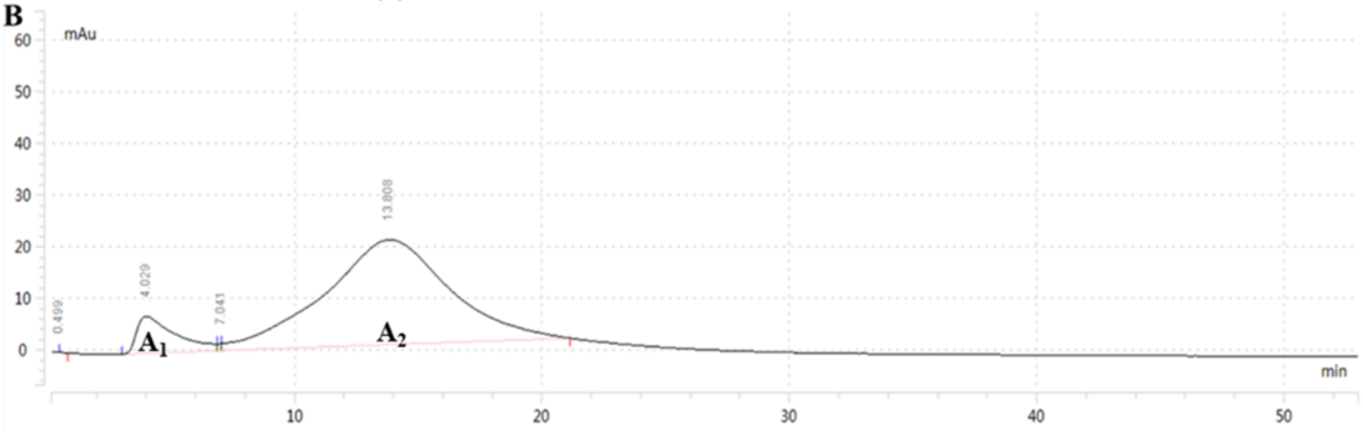

Supplementary Figure S3 Isolation and purification of agarase in the medium after cultivation of *V. fluvialis* A8 using **(A)** ammonium sulfate precipitation and **(B)** DEAE anion exchange chromatography. The eluent collected from protein peak A_2 that showed good agarase activity was used for the study of agarase properties. **(C)** Changes of total protein (mg), total agarase activity (U), specific activity (U/mg), recovery (%) and purification fold after isolation and purification.
