# Supplementary material for: Comparative Genomic and Secretomic Analysis Provide Insights Into Unique Agar Degradation Function of Marine Bacterium Vibrio fluvialis A8 Through Horizontal Gene Transfer
Source: Front Microbiol. 2020 Aug 11;11:1934. doi: 10.3389/fmicb.2020.01934 (PMC7432431; doi:10.3389/fmicb.2020.01934)
Supplement: Supplementary file 4 [file Table_1.DOCX]

Supplementary Material

**Supplementary Table S1** Physiological and biochemical characteristics of *V. fluvialis* A8 tested by VITEK 2 GN system

| Abbr. | Reaction | Result | Abbr. | Reaction | Result |
| --- | --- | --- | --- | --- | --- |
| APPA | Ala-Phe-Pro-arylamidase | + | SAC | Saccharose/Sucrose | + |
| ADO | Adonitol | - | dTAG | D-tagatose | - |
| PyrA | L-pyrrolydonyl-arylamidase | + | dTRE | D-trehalose | + |
| 1ARL | L-arabitol | - | CIT | Citrate(sodium) | + |
| dCEL | D-cellobiose | - | MNT | Malonate | - |
| BGAL | Beta-galactosidase | + | 5KG | 5-keto-D-gluconate | - |
| H_2_S | H_2_S production | - | 1LATK | L-lactate alkalinisation | + |
| BNAG | Beta-N-acetyl-glucosaminidase | + | AGLU | Alpha-glucosidase | - |
| AGLTp | Glutamyl arylamidase pNA | - | SUCT | Succinate alkalinisation | + |
| dGLU | D-glucose | + | NAGA | Beta-N-acetyl-galactosamanidase | - |
| GGT | Gama-glutamyl-transferase | - | AGAL | Alpha-galactosidase | + |
| OFF | Fermentation/glucose | + | PHOS | Phosphate | + |
| BGLU | Beta-glucosidase | - | GlyA | Glycine arylamidase | + |
| dMAL | D-maltose | + | ODC | Ornithine decarboxylase | - |
| dMAN | D-mannitol | + | LDC | Lysine decarboxylase | - |
| dMEN | D-mannose | + | 1HISa | L-histidine assimilation | - |
| BXYL | Beta-xylosidase | - | CMT | Coumarate | + |
| BALap | Beta-alanine arylamidase pNA | - | BGUR | Beta-glucoronidase | - |
| ProA | L-proline arylamidase | + | 0129R | 0/129 resistance | - |
| LIP | Lipase | - | GGAA | Glu-Gly-Arg-arylamidase | - |
| PLE | Palatinose | - | 1MLTa | L-malate assimilation | - |
| TyrA | Tyrosine arylamidase | + | ELLM | Ellman | + |
| URE | Urease | + | 1LAT | L-lactate assimilation | + |
| dSOR | D-sorbitol | - |  |  |  |
