# Supplementary material for: Comparative Genomic and Secretomic Analysis Provide Insights Into Unique Agar Degradation Function of Marine Bacterium Vibrio fluvialis A8 Through Horizontal Gene Transfer
Source: Front Microbiol. 2020 Aug 11;11:1934. doi: 10.3389/fmicb.2020.01934 (PMC7432431; doi:10.3389/fmicb.2020.01934)
Supplement: Supplementary file 5 [file Table_2.DOCX]

Supplementary Material

**Supplementary Table S2** Peptides of agarase (Protein 3152) in the secretome of *V. fluvialis* A8 tested by Label‑free quantitative LC–MS/MS

| No. | Annotated Sequence | Qvality PEP | Qvality q-value | Positions in Protein 3152 | Sequence Length | RT [min] | Abundances |
| --- | --- | --- | --- | --- | --- | --- | --- |
| 1 | [K].QGEGVSFYANNIDAR.[H] | 9.93E-05 | 0 | [529-543] | 15 | 37.7710 | 164289332 |
| 2 | [K].SLNWQFANK.[Y] | 0.03 | 0 | [734-742] | 9 | 39.6015 | 102389626 |
| 3 | [K].IYAPNSWNWSDQK.[E] | 1.86E-04 | 0 | [74-86] | 13 | 45.3586 | 12929400 |
| 4 | [R].MTDTYTFTGESSTMPSDVR.[K] | 1.09E-03 | 0 | [486-504] | 19 | 31.1364 | 70384301 |
| 5 | [R].VSDGTTEPTPPPTDPEDVSGR.[V] | 2.33E-06 | 0 | [176-196] | 21 | 24.5666 | 9232439 |
| 6 | [K].YIPANSTQTVYVSVK.[D] | 3.36E-04 | 0 | [301-315] | 15 | 34.3849 | 75807821 |
| 7 | [K].YFEVVK.[Q] | 0.21 | 0.005 | [743-748] | 6 | 27.9370 | 126510876 |
| 8 | [K].TLLQLETFEELTDSIEATGADIK.[L] | 1.25E-03 | 0 | [204-226] | 23 | 58.9264 | 2911972 |
| 9 | [R].SQYGGAPAGSSIVADQDCK.[L] | 2.09E-06 | 0 | [431-449] | 19 | 24.4047 | 46516963 |
| 10 | [K].VHADVVSFNIYK.[D] | 8.80E-04 | 0 | [780-791] | 12 | 39.3479 | 72102063 |
| 11 | [K].DLNDVSAYEELVDSLGQNNQHDFYAK.[L] | 1.00E-08 | 0 | [381-406] | 26 | 58.0258 | 42143567 |
| 12 | [R].FADWGR.[T] | 0.24 | 0.006 | [765-770] | 6 | 27.5738 | 47085928 |
| 13 | [R].EKLDGLGAR.[E] | 0.02 | 0 | [411-419] | 9 | 13.1975 | 84789612 |
| 14 | [K].ELVFDVVNNTNEDINYGVK.[I] | 4.47E-06 | 0 | [87-105] | 19 | 55.9641 | 1386688 |
| 15 | [K].EMGPVHSGPVK.[Q] | 5.00E-03 | 0 | [518-528] | 11 | 12.9838 | 52682519 |
| 16 | [R].ETQLLGK.[L] | 0.24 | 0.006 | [420-426] | 7 | 13.8823 | 5934102 |
| 17 | [K].AAAANMPYVANGWVLHHETSENPVNR.[I] | 1.86E-04 | 0 | [578-603] | 26 | 43.1381 | 2768075 |
| 18 | [K].AAFIWFLENGFGQSK.[T] | 6.04E-05 | 0 | [683-697] | 15 | 58.7502 | 7827453 |
| 19 | [K].AEFPNNLYLGAR.[F] | 3.15E-03 | 0 | [753-764] | 12 | 46.5572 | 44731107 |
| 20 | [K].ATFNNAWSSVK.[L] | 0.05 | 0 | [241-251] | 11 | 35.4623 | 78616906 |
| 21 | [R].AISDIVNTLGMR.[E] | 7.34E-04 | 0 | [319-330] | 12 | 54.8898 | 129484242 |
| 22 | [K].ILTNYVWDDANALSEYFNVPANTTLK.[D] | 8.75E-06 | 0 | [106-131] | 26 | 58.6959 | 7878454 |
| 23 | [R].IGTGYWGPIADPFDPNFALAAK.[K] | 1.59E-04 | 0 | [604-625] | 22 | 58.5541 | 4907450 |
| 24 | [K].LIDEGVSIGETALK.[A] | 4.07E-04 | 0 | [227-240] | 14 | 44.6259 | 70214480 |
| 25 | [R].LINCELYGDDCSALEANTETR.[S] | 3.01E-06 | 0 | [903-923] | 21 | 42.6940 | 2969036 |
| 26 | [R].HGGEEAWQDITIK.[R] | 7.50E-06 | 0 | [544-556] | 13 | 38.1049 | 10497993 |
| 27 | [R].HGGEEAWQDITIKR.[M] | 2.80E-04 | 0 | [544-557] | 14 | 31.8880 | 2249606 |
